# Imported SARS-CoV-2 Variant P.1 in Traveler Returning from Brazil to Italy

**DOI:** 10.3201/eid2704.210183

**Published:** 2021-04

**Authors:** Fabrizio Maggi, Federica Novazzi, Angelo Genoni, Andreina Baj, Pietro Giorgio Spezia, Daniele Focosi, Cristian Zago, Alberto Colombo, Gianluca Cassani, Renee Pasciuta, Antonio Tamborini, Agostino Rossi, Martina Prestia, Riccardo Capuano, Lorenzo Azzi, Annalisa Donadini, Giuseppe Catanoso, Paolo Antonio Grossi, Lorenzo Maffioli, Gianni Bonelli

**Affiliations:** ASST Sette Laghi, Varese, Italy (F. Maggi, F. Novazzi, A. Baj, C. Zago, A. Colombo, G. Cassani, R. Pasciuta, A. Tamborini, A. Rossi, M. Prestia, R. Capuano, L. Azzi, P.A. Grossi, L. Maffioli, G. Bonelli);; University of Insubria, Varese (F. Maggi, A. Genoni, A. Baj, P.A. Grossi);; University of Pisa, Pisa, Italy (P.G. Spezia);; North-Western Tuscany Blood Bank, Pisa (D. Focosi);; ATS Insubria, Varese (A. Donadini, G. Catanoso)

**Keywords:** respiratory infections, severe acute respiratory syndrome coronavirus 2, SARS-CoV-2, SARS, COVID-19, coronavirus disease, zoonoses, viruses, variant of concern, P.1, B.1.1.28, 20J/501Y.V3, Brazil, Italy

## Abstract

We report an imported case of severe acute respiratory syndrome coronavirus 2 (SARS-CoV-2) variant P.1 detected in an asymptomatic traveler who arrived in Italy on an indirect flight from Brazil. This case shows the risk for introduction of SARS-CoV-2 variants from indirect flights and the need for continued SARS-CoV-2 surveillance.

Severe acute respiratory syndrome coronavirus 2 (SARS-CoV-2) variant P.1 currently is causing a major outbreak of coronavirus disease (COVID-19) in the Amazonas province of Brazil (N.R. Faria et al., unpub. data, https://virological.org/t/genomic-characterisation-of-an-emergent-sars-cov-2-lineage-in-manaus-preliminary-findings/586). The P.1 variant also is known as B.1.1.28 in the Phylogenetic Assignment of Named Global Outbreak Lineages (https://cov-lineages.org/pangolin.html) and as 20J/501Y.V3 in NextStrain (https://nextstrain.org). Preliminary reports have associated several spike protein mutations harbored in the P.1 variant with escape from neutralizing monoclonal antibodies (mAb) and P.1 was detected in convalescent serum collected during previous epidemic waves (Z. Liu et al., unpub. data, https://www.biorxiv.org/content/10.1101/2020.11.06.372037v1; S. Jangra et al., unpub. data, https://www.medrxiv.org/content/10.1101/2021.01.26.21250543v1).

The B.1.1.28 lineage emerged in Brazil during February 2020, and 2 subclades recently evolved separately (C.M. Voloch et al., unpub. data, https://doi.org/10.1101/2020.12.23.20248598; N.R. Faria, et al., unpub. data, https://virological.org/t/genomic-characterisation-of-an-emergent-sars-cov-2-lineage-in-manaus-preliminary-findings/586). During January 2021, SARS-CoV-2 variant P.1 was reported in 4 travelers returning to Japan from Amazonas state in Brazil (*1*). The strain identified in the travelers was associated with E484K, K417N, and N501Y mutations as noted in the the B.1.351 line 20I/501.V2 clade of South African lineage (*1*). In addition, 1 case of reinfection has been documented months after a B.1 primary infection (F. Naveca et al., unpub. data, https://virological.org/t/sars-cov-2-reinfection-by-the-new-variant-of-concern-voc-p-1-in-amazonas-brazil/596). Another lineage, P.2, was reported in Rio de Janeiro, Brazil, but has been associated with spike mutations only in E484K; >2 cases of reinfection have been documented several months after primary B.1.1.33 infections (P. Resende et al., unpub. data, https://virological.org/t/spike-e484k-mutation-in-the-first-sars-cov-2-reinfection-case-confirmed-in-brazil-2020/584; C.K. Vasques Nonaka et al., unpub. data, https://doi.org/10.20944/preprints202101.0132.v1). Among the spike mutations, E484K is considered the main driver of immune evasion to mAbs and convalescent serum (A.J. Greaney et al., unpub. data, https://doi.org/10.1101/2020.12.31.425021). Of note, many of the most potent mRNA vaccine-elicited mAbs were 3- to 10-fold less effective at neutralizing pseudotyped viruses carrying E484K (K. Wu et al., unpub. data, https://doi.org/10.1101/2021.01.25.427948), which has unknown implications for protection. We report an asymptomatic traveler from Brazil who tested positive for the SARS-CoV-2 P.1 variant in a screening nasopharyngeal swab sample. 

After visiting São Paulo, Brazil, during November 23, 2020–January 16, 2021, a family, including a 33-year-old man, his 38-year-old wife, and his 7-year-old daughter, flew back to their home in Italy. During their time in Brazil, the family did not travel outside of São Paulo, which is >2,000 miles from Amazonas. The family took an indirect return flight; they flew from São Paulo/Guarulhos International Airport in Brazil to Madrid, Spain, and from there flew to Milan Malpensa Airport in Italy. Molecular tests were performed on all 3 family members at the departure airport in Brazil, and all were SARS negative. 

The family arrived in Milan on the afternoon of January 17 and took a train and a car to their home, 30 miles from Milan. Under current recommendations in Italy, all persons entering the country can decide to be screened for SARS-CoV-2. After consulting a general practitioner on January 21, the father went to the hospital for a screening nasopharyngeal swab sample. The sample was tested by using the Alinity platform (Abbott, https://www.abbott.com), which returned a positive result for SARS-CoV-2 RNA with a cycle threshold of 23. Reverse transcription PCR (RT-PCR) fragments corresponding to the receptor-binding domain (RBD) in the spike gene of SARS-CoV-2 were amplified from purified viral RNA by using a OneStep RT-PCR Kit (QIAGEN, https://www.qiagen.com). We used a reference sequence from GSAID (https://www.gisaid.org; accession no. EPI_ISL_402124) and nucleotide sequences of primer sets to map genome locations (Figure; Appendix). The sequence of RBD from the patient included the P.1 barcoding mutations K417T, E484K, and N501Y. We deposited these data in GenBank (accession no. MW517286) and GISAID (accession no. EPI-ISL-869166).

**Figure:**
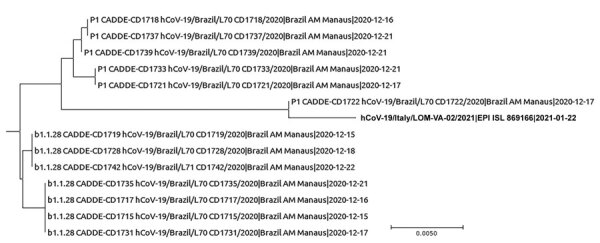
Phylogenetic tree of severe acute respiratory syndrome coronavirus 2 variant P.1 sequences from a male traveler returning from Brazil to Italy and reference sequences from Brazil. Bold text indicates sequence from the traveler. Scale bar indicates nucleotide substitutions per site.

SARS-CoV-2 variant P.1 is characterized by K417N, but K417T also has been reported in several cases before our patient (*1*), suggesting ongoing evolution. On January 22, 2021, after we reported the sequencing results, the patient was admitted to the infectious and tropical diseases unit of ASST dei Sette Laghi–Ospedale di Circolo e Fondazione Macchi (Varese, Italy) for observation. The patient remained asymptomatic and was discharged on January 29. The patient’s spouse also tested positive for SARS-CoV-2 RNA via a nasopharyngeal swab sample. Antibody tests conducted by using Liaison Analyzer (DiaSorin, https://www.diasorin.com) were negative for SARS-CoV-2 S1/S2 IgG in serum of both the man and his wife, suggesting a primary infection.

Direct flights from Brazil to Italy were canceled upon the unilateral decision of the government of Italy on January 16, 2021, but our findings confirm the risk for introducing of SARS-CoV-2 variants from indirect flights if no surveillance measures are implemented at arrival. This case also suggests wider circulation of SARS-CoV-2 variant P.1 in areas other than Amazonas in Brazil. P.1-specific primer sets recently have been designed (A. Lopez-Rincon et al., unpub. data, https://doi.org/10.1101/2021.01.20.427043) and will aid in development of large-scale screening programs for this variant.

Appendix Sample preparation and sequencing of RNA from severe acute respiratory syndrome coronavirus 2 variant P.1 detected in a traveler returning from Brazil to Italy.
