# Supplementary material for: Imported SARS-CoV-2 Variant P.1 in Traveler Returning from Brazil to Italy
Source: Emerg Infect Dis. 2021 Apr;27(4):1249–51. doi: 10.3201/eid2704.210183 (PMC8007292; doi:10.3201/eid2704.210183)
Supplement: Appendix — Sample preparation and sequencing of RNA from severe acute respiratory syndrome coronavirus 2 variant P.1 detected in a traveler returning from Brazil to Italy. [file 21-0183-Techapp-s1.pdf]

Article DOI: https://doi/org/10.3201/eid2704.210183

Imported SARS-COV-2 Variant P.1 Detected in Traveler Returning from Brazil to Italy

Appendix

Supplementary Methods

Reverse transcription PCR (RT-PCR) fragments corresponding to the receptor-binding domain (RBD) in the spike gene of SARS-CoV-2 were amplified from purified viral RNA collected from the patient's nasophyringeal swab by using a OneStep RT-PCR Kit (QIAGEN, https://www.qiagen.com). We performed 2 nested PCR (nPCR) reactions the amplified viral RNA, nPCRA and nPCRB, in 50 µL according to the manufacturer's instructions. Amplification conditions were 50°C for 30 min followed by 94°C for 15 min plus 40 cycles of 94°C for 30 s, 60°C for 30 s, and 72°C for 1 min with a final extension step of 72°C for 10 min. After the first PCR reaction, we used 5 µL of amplified product for the second nPCR reaction. Amplification conditions for nPCRB were 95°C for 3 min plus 30 cycles of 95°C for 30 s, 60°C for 30 s, and 72°C for 1 min with a final extension step of 72°C for 10 min. We purified the amplified products by using QIAquick PCR Purification kit (QIAGEN) to prepare for Sanger sequencing analysis. We included negative RNA samples and template controls in every assay and found all to be negative. We used BigDye Terminator v.1.1 (Applied Biosystems, https://www.thermofisher.com) cycle sequencing kit to sequence the purified RT-PCR products. We purified the sequencing reactions by using Centri-Sep Spin Columns (Princeton Separations, Inc., https://www.prinsep.com) and analyzed on a SeqStudio Genetic Analyzer (Applied Biosystems). We identified sequence variants by using CLC main workbench 7.0.0 (QIAGEN). We used reference sequence GSAID accession no. EPI_ISL_402124 and nucleotide sequences of primer sets to map genome locations (Appendix Table) The sequence of receptor binding domain from the patient included the P.1 barcoding mutations K417T, E484K, and N501Y. We

deposited these data in NCBI (https://www.ncbi.nlm.nih.gov/genbank; GenBank accession no. MW517286) and GISAID (https://www.gisaid.org; GISAID accession no. EPI-ISL-869166).

Appendix Table. List of oligonucleotide primers used for amplification of severe acute respiratory syndrome coronavirus 2 receptor-binding domain*

nPCR	Name	Sequence, 5′–3′	Genome location, nt†	Amplicon size, bp
A	RBD_F1	GTACGTTGAAATCCTTCACTGTAGA	22464–22488	936
	RBD_R1	GATAAAGAACAGCAACCTGGTTAGAAG	23399–23373	
B	RBD_F2	CAAACTTCTAACTTTAGAGTCCAACC	22502–22527	863
	RBD_R2	CCTGGTGTTATAACACTGACACCA	23364–23341	

*bp, base pairs; nPCR, nested PCR; nt, nucleotide.
†Genome location within the hCoV-19/Wuhan/WIV04/2019 genomic sequence (GSAID accession no. EPI_ISL_402124).
